# Identification and validation of mitochondrial-related genes in intestinal ischemia-reperfusion injury based on WGCNA and machine learning

**DOI:** 10.3389/fmolb.2025.1691749

**Published:** 2025-10-29

**Authors:** YiChen Hu, Jie Huang, XiaoLi Min, YuanPei Zhao, JiaHui Wang, HongYuan Liu, KaiWen Shi, WenLiang Li, WeiMing Li

**Affiliations:** ^1^ Department of Gastrointestinal Surgery, Second Affiliated Hospital of Kunming Medical University, Kunming, Yunnan, China; ^2^ Department of Hepatobiliary and Pancreatic Surgery, Second Affiliated Hospital of Kunming Medical University, Kunming, Yunnan, China; ^3^ Department of Psychiatric Sleep Medicine Center, Second Affiliated Hospital of Kunming Medical University, Kunming, Yunnan, China; ^4^ Yunnan Cancer Hospital, Kunming, Yunnan, China

**Keywords:** intestinal ischemia-reperfusion (II/R) injury, mitochondria, WGCNA, bioinformatics analysis, machine learning

## Abstract

**Background:**

Severe ischemia-reperfusion (II/R) injury of the intestines is a leading cause of death and disability. According to earlier research, modulating mitochondrial function is the primary mechanism by which II/R injury is ameliorated. In order to further molecular diagnostics and discover possible treatment targets, it is essential to find biomarkers of mitochondria in II/R injury.

**Methods:**

The datasets GSE96733 and GSE37013, along with mitochondrial-related genes (MRGs), were obtained from the Gene Expression Omnibus (GEO) database and MitoCarta3.0, respectively. GSE96733 conducted differential expression gene (DEGs) analysis and weighted gene co-expression network analysis (WGCNA) module screening. In order to find MRGs that were expressed differently, we got their intersection (DEMRGs) and gene enrichment analysis was carried out. The hub genes were screened using machine learning approaches, protein-protein interaction (PPI) network analysis, and Molecular Complex Detection (MCODE). A nomogram was developed for diagnostic evaluation. Furthermore, the relationship between hub gene expression profiles and immune infiltration landscapes was interrogated through immune cell infiltration analysis. The expression patterns of the hub genes were further validated in the II/R injury model through dataset validation and qRT-PCR assays. The procedure concluded in a gene-related hub network, DSigDB prediction of prospective therapeutic compounds, and molecular docking simulations of the drugs’ binding affinity with important target proteins.

**Results:**

Hub genes have been found in five different DEMRGs: Pdk4, Yrdc, Bcl2l11, Bcl2a1d and Pmaip1. The nomogram model was beneficial for diagnosis. Dendritic cells (DC) and M2 macrophages are strongly linked to the 5 Hub genes, according to immune cell infiltration research. Afterwards, the regulatory network showed that hub genes and miRNAs had a complicated connection. Additionally, securinine and ABT-737 were anticipated to be possible therapeutic agents for II/R injury. The validation results for the four hub genes (Pdk4, Yrdc, Bcl2l11, and Pmaip1), obtained from both independent datasets and qRT-PCR, were consistent with the initial bioinformatics analysis.

**Conclusion:**

Pdk4, Yrdc, Bcl2l11, and Pmaip1 have been identified as hub genes closely associated with mitochondrial function in eraly II/R injury, thereby providing a theoretical basis for the diagnosis and treatment of eraly II/R injury.

## 1 Introduction

Intestinal ischemia-reperfusion (II/R) injury commonly occurs in various clinical settings, including abdominal vascular surgery, small intestine transplantation, severe hemorrhagic shock, and strangulated intestinal obstruction (Xu et al., 2023). This mechanism can cause intestinal mucosal cells to die and bleed, and it can also set off systemic inflammatory response syndrome (SIRS) and multiple organ dysfunction syndrome (MODS) (Wu et al., 2013; Zhu et al., 2017; Sun et al., 2018). II/R injury involves oxidative stress, inflammation, apoptosis, and intestinal barrier dysfunction (Lin et al., 2021). Oxidative stress is one of the main causes of II/R injury, and an important aspect that makes the damage worse is the overproduction of reactive oxygen species (ROS) (Sasaki and Joh, 2007). The severity of injury is regulated by the activation of key signaling routes, including Nrf2, Wnt/β-catenin, and the PI3K/Akt cascade (Li G. et al., 2021). Furthermore, research has shown that Bryostatin-1 can activate the Nrf2/HO-1 signaling pathway. This pathway helps to reduce oxidative stress and intestinal barrier dysfunction caused by II/R injury, lending credence to this viewpoint (Liu et al., 2023). Apoptosis represents a key mechanism contributing to cellular demise in II/R injury (Zu et al., 2016). Attenuating II/R injury may be accomplished through inhibition of pro-apoptotic factors such as Bax and caspase-3/9, or conversely, by enhancing the activity of anti-apoptotic proteins like Bcl-2 (Wen et al., 2019; Almoiliqy et al., 2020). Despite favorable outcomes in the treatment of II/R injury via antioxidant, anti-inflammatory, and anti-apoptotic mechanisms (Wang et al., 2021), there are currently limited alternatives for detecting and treating this condition. Therefore, finding important biomarkers and researching new treatment approaches is crucial. An integral part of every eukaryotic cell is the mitochondria, which are in charge of the cell’s energy metabolism and the production of adenosine triphosphate (ATP) via oxidative phosphorylation. The mitochondria are not only responsible for generating energy, but they also have a role in regulating the homeostasis of calcium ions, apoptosis, inflammatory reactions, and immunological modulation (Tábara et al., 2025). Several diseases, such as metabolic disorders, neurological diseases, and cardiovascular diseases, are linked to dysregulation of mitochondrial activity (Chernega et al., 2022). There are many avenues by which mitochondria, which are essential for cellular energy metabolism, contribute to the onset and progression of II/R injury (Bock and Tait, 2020). Among the novel approaches to treating II/R injury in recent years has been the focus on regulating mitochondrial function. Metformin decreases the formation of mitochondrial-associated membranes (MAM) (Zhao et al., 2025), mitophagy clears damaged mitochondria (Li S. et al., 2021), and dexmedetomidine modulates mitochondrial stability (Wu et al., 2024), all of which demonstrate certain protective effects. Wang found that Mitochondrial Replenishment Therapy (MRT) is a potentially useful treatment since it restores the energy metabolism function of damaged cells by transplanting healthy mitochondria into them (Wang J. et al., 2025). However, the mitochondrial-related genes involved in the pathogenesis of II/R injury remain largely unclear. So, new possible biomarkers for II/R injury may be discovered by applying bioinformatics to studies of mitochondrial-related biomarkers. The immune system plays a crucial role in the development of II/R injury (Zhang F. L. et al., 2023). According to recent research, lymphocytes—particularly T cells and, to a lesser degree, B cells—play a crucial role in II/R damage. Despite this, in-depth studies investigating the biological functions of MRGs and the relationship between MRG expression levels and immune cell infiltration following II/R injury are few (Linfert et al., 2009). A lot of attention has been directed towards non-coding RNAs (circRNA, miRNA, lncRNA, etc.) as of late (Zhang J. et al., 2023). The therapeutic inhibition of microRNA-122a was found to activate the EGFR-NLRP3 signaling axis, a mechanism that ultimately leads to reduced pyroptosis and an alleviation of II/R injury. These findings highlight the importance of miRNA-mediated regulation in the context of II/R injury (Wang et al., 2022).

There has been a lot of research looking at the role of mitochondria as regulators in II/R injury, but most of it has ignored the immune microenvironment and how non-coding RNAs affect target gene expression, focusing instead on the biological functions carried out by individual genes and how mitochondrial function is regulated. Hence, we used biological analysis to filter hub genes linked to immune infiltration and mitochondrial function, and we gathered II/R damage information from public databases. Using these hub genes as nodes, we built a circRNA-miRNA-mRNA regulatory network and used it to forecast the diagnosis of II/R injury. In order to assess the binding affinity of medications with important target proteins, we conducted molecular docking simulations and also projected prospective therapeutic molecules. Finally, qRT-PCR validation provided new insights into the etiology of II/R injury and underscored the critical role of these hub genes.

## 2 Materials and methods

### 2.1 Data used

**FIGURE 1:**
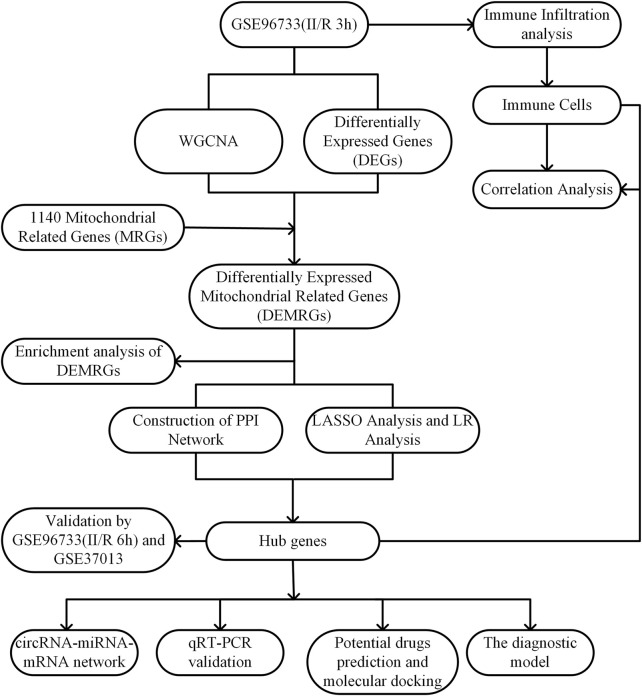
Experimental design Roadmap.

The raw data for GSE96733 are available from the GEO database (http://www.ncbi.nlm.nih.gov/geo/), based on the GPL23038 platform (Clariom S Mouse) using the Affymetrix Clariom S Mouse assay. This dataset, designated as II/R 3h, comprises four sham-operated controls and four intestinal ischemia/reperfusion (II/R) samples collected at 3 hours post-operation. Differential expression analysis between sham and II/R groups was conducted with the “limma” package in R, applying thresholds of |log_2_FC| ≥ 1 and an adjusted P-value <0.05. Resulting differentially expressed genes were visualized via volcano plots and heatmaps generated with the “ggplot2” package in R. For validation, the GSE96733 (II/R 6 h) subset—including four sham and four II/R samples at six hours—was employed. Additional confirmation was obtained using the human dataset GSE37013 from GEO, which contains 21 samples profiled on the Illumina HumanHT-12 V3.0 beadchip platform (GPL6947), including seven sham controls, seven samples at 30 min post-reperfusion (II/R 30 min), and seven at 120 min post-reperfusion (II/R 120 min). The overall experimental workflow is summarized in Figure 1.

### 2.2 Weighted gene co-expression network analysis (WGCNA)

To identify co-expression modules associated with II/R injury, we performed weighted gene co-expression network analysis (WGCNA) on the GSE96733 (II/R 3 h) dataset using the “WGCNA” R package. The optimal soft threshold was selected with the “pickSoftThreshold” function to ensure a scale-free network topology. A weighted adjacency matrix was then constructed based on the Pearson correlation coefficient, from which a topological overlap matrix (TOM) was derived. Modules consisting of genes with similar expression patterns were identified with a minimum size of 30 and a merge cut height of 0.25. From these modules, genes demonstrating the strongest association with II/R injury were subsequently extracted for further analysis (Zeng et al., 2023).

### 2.3 Identification and functional enrichment of differentially expressed mitochondrial-related genes

A total of 1,140 mitochondrial-related genes (MRGs) were obtained from the MitoCarta3.0 datasets (http://www.broadinstitute.org/mitocarta). Next, we used Xiantao Academic (https://www.xiantaozi.com/). It selected the most pertinent modules in WGCNA, combined MRGs with DEGs, and finally found the differentially expressed mitochondrial-related genes (DEMRGs). GO and KEGG pathway enrichment were performed with the assistance of the package named clusterProfiler using R software (version 4.2.2) (Wu et al., 2021). Gene Ontology (GO) categorization is divided into three primary domains: namely, Biological Process (BP), Molecular Function (MF), and Cellular Component (CC). By changing the P-value level to 0.05 and below, appropriate GO keywords and signal pathways were estimated. Subsequently, the results were plotted with the help of R packages “ggplot2” and “GOplot” (Walter et al., 2015). We performed a t-test on DEMRGs expression levels extracted to test the hypothesis that expression levels are not likely to be the same in the GSE96733 (II/R 3 h) dataset. The “ggpubr” tool in R software was used to construct a violin plot, which illustrated the differential expression of DEMRGs. We utilized the R package clusterProfiler for our calculations and applied the Benjamini–Hochberg (BH) method to adjust the raw p-values for multiple testing correction, thereby controlling the false discovery rate (FDR). Only biological processes or signaling pathways with an adjusted FDR <0.05 are considered statistically significant. This method effectively reduces the risk of false positives associated with multiple comparisons, ensuring the stability and reliability of the enrichment results.

### 2.4 PPI network analysis of DEMRGs

To explore the underlying links between DEMRGs with an interaction score larger than 0.4, protein-protein interaction (PPI) network analysis was conducted using the STRING online database (https://cn.string-db.org/). Subsequently, the filtered network was examined and graphically represented employing Cytoscape software (version 3.10.3). The first step was to find mitochondria-related hub genes using the MCODE algorithm. An technique developed for the purpose of finding chemical complexes or highly linked modules within PPI networks, MCODE (Molecular Complex Detection) is based on network clustering. It finds subnetworks with high levels of interaction by studying the network’s topology, mainly by determining the connection scores of nodes (like proteins) to locate “high-density regions” (Zhou et al., 2021). The hub genes were also computed using CytoHubba using two distinct algorithms, Degree and MCC. Genes with many direct connections to other nodes in the network, or a high Degree value, are usually located at the center of the network and have important regulatory or biological roles (Eisele et al., 2021). Largest Clique Centrality (MCC) determines a node’s network significance by finding its position within the largest clique. The most extensive group of completely linked nodes in a network is called a maximum clique. According to Bhattacharyya et al. (2023), a node’s involvement in interacting sub-networks is directly correlated with its MCC score. A node with a high MCC score may play a crucial role in regulating the network’s stability or functionality. (Bhattacharyya et al., 2023).

### 2.5 Machine learning and identifying hub genes

We utilized the Lassenet alpha algorithm, a logistic regression method for variable selection that improves predictive accuracy, to identify the core genes and build a diagnostic model for II/R damage (Friedman et al., 2010). One of the most well-known probabilistic classification algorithms, Logistic Regression (LR) uses the Sigmoid function to transform the output of linear regression into a probability of the target category on the interval (0,1) (Thabtah et al., 2019). Python is used to display the outcomes of both approaches. Clarification of selection terminology and data-driven thresholds. All predictors were z-score standardized prior to modeling. For logistic regression, we report absolute standardized coefficients (not an undefined “feature importance” metric). The value 0.18 was used as a screening heuristic, motivated by the elbow/upper-quartile region of the rank-ordered absolute standardized coefficients observed during cross-validation. For Lasso, we selected the λ.1se solution and report non-zero coefficients at that setting; the value 0.045 corresponds to the smallest non-zero coefficient under λ.1se across folds and served as a data-driven floor to exclude borderline, fold-specific entries. These magnitudes are not interpreted as significance thresholds; statistical inference for LR is based on standardized coefficients with two-sided Wald tests and Benjamini–Hochberg false discovery rate adjustment. Hub candidates were then summarized alongside PPI evidence as corroborative (not exclusionary) information.

### 2.6 Construction and verification of the diagnostic model

Depending on the five hub genes, a nomogram was drawn with assistance of the rms software (Zhao and Li, 2023). Their sensitivity and specificity were calculated using the pROC R program to determine the area under the receiver operating characteristic (ROC) curve that was used to analyze the diagnostic performance of these hub genes in terms of identifying II/R injury (Hanley and McNeil, 1982). To provide additional support, the GSE37013 validation set was employed to confirm the expression levels of the five hub genes; the accuracy of each gene’s diagnostic value was subsequently examined using ROC curves.

### 2.7 Confirmation of hub gene expression patterns using independent datasets

Gene expression data for key II/R injury-related genes were obtained from the GSE96733 (II/R 6 h) and GSE37013 datasets and subjected to statistical analysis using t-tests. The expression profiles of the hub genes were assessed and visualized through violin plots, which were generated with the “ggplot2” package in R.

### 2.8 Immune cell in filtration analysis

To characterize immune cell infiltration dynamics, the CIBERSORT algorithm was employed to evaluate shifts in immune composition. This study aimed to characterize the relative proportions of infiltrating immune cells following II/R injury. The infiltration levels of 25 distinct immune cell types in mice were visualized using the “ggcorrplot” package (Chen et al., 2017).

### 2.9 II/R injury model construction

Although the mice received water *ad libitum*, they had to fast for 12 h before the establishment of a mouse II/R injury model. Anesthesia was produced by 30 mg/kg pentashock manually intraperitoneally. The mice were put in sterile material and straps as we observed the signs of weakness in their legs. There was a thorough cleaning up and disinfection of the abdominal skin. A 1.5 cm midline abdominal incision was made approximately 2 mm below the xiphoid process following local anesthesia with 2% lidocaine. The SMA was identified and dissected after having accessed the abdominal cavity in a systematic manner that allowed exposing layer by layer the abdominal cavity and the intestinal tract. Time was registered a few moments later by occluding SMA and its branches using a non-traumatic microvascular clamp. Recall guidance to ischemia was successful when blood flow was absent through the arteries and intestinal swelling with a pale color. To minimize moisture loss, the intestine was carefully repositioned within the abdominal cavity and covered with a saline-moistened gauze. Reperfusion was initiated 45 min later by removal of the microvascular clamp. The effective reperfusion was shown by the progressive increase in arterial pulse and the recovery of the intestine’s brilliant red color. The next step was to inject 0.5 mL of warmed saline into the abdomen, and then to stitch the abdomen in layers using 3–0 silk thread. The next step was to move the mice to a warmer environment by placing their cage close to a heater. Following a 3-h reperfusion period, all animals were humanely euthanized via intraperitoneal administration of pentobarbital sodium at a dosage of 200 mg/kg. The next step was to extract the small intestinal tissue and store it at a temperature of −80 °C. For the sham surgery group, the previous steps were just repeated, except isolating the superior mesenteric artery, which was not occluded.

### 2.10 Reverse transcription-quantitative PCR (RT-qPCR)

Using a Lifetech 15596026 RNA extraction kit, total RNA was isolated from frozen intestinal tissue. After that, a cDNA synthesis kit (FastKing RT Kit FastKing cDNA KR116) was used for reverse transcription. Relative mRNA expression levels were quantified on a Roche LightCycle 96 real-time PCR system using the 2^−ΔΔCq^ method, with β-actin serving as the internal reference. The corresponding primer sequences are provided in Table 1.

**TABLE 1:** Primer sequences used in the qRT-PCR experiments.

Gene	Forward primer (5′–3′)	Reverse primer (3′–5′)
β-actin	TATGGAATCCTGTGGCATC	GTGTTGGCATAGAGGTCTT
Bcl2l11	GACAAGTCAACACAAACC	ATTCTCCAACTGATCCATT
Bcl2a1d	AACTTCCACAAGAGCAGAT	ATTCCGCCGTATCCATTC
Pmaip1	AAGGACGAGTGTGCTCAA	GCTTGGAAATCAAATTCAGAAGTT
Yrdc	GTCACCTCTGTCATCGTA	ATGCTTCACCTTCTGGAT
Pdk4	AACTGTGATGTGGTAGCA	CTGGCGATGTTAGATAATACTG

### 2.11 Construction of the circRNA-miRNA-mRNA regulatory network

We are primarily concerned with translating the genetic studies conducted on mice to people. Accordingly, human orthologs of the hub genes were identified by mapping homologous genes through two resources: the “homologene” R package and the online platform provided by Xiantao Academic (https://www.xiantaozi.com/). As a first step in conducting a comprehensive analysis of the miRNA regulatory network, we predicted and screened the relationships between miRNAs and their probable target mRNAs and circRNAs using the ENCORI database (https://rnasysu.com/encori/). Enhancing the trustworthiness of the projected results, ENCORI offers bioinformatics support based on high-throughput sequencing data. This support allows for the discovery of miRNA-mRNA and miRNA-circRNA binding sites, which have been validated by CLIP-seq investigations. We next used Cytoscape to show a network consisting of circRNA-miRNA-mRNA ceRNA (competing endogenous RNA), which we constructed by integrating the screened miRNA-mRNA and miRNA-circRNA regulatory pairs.

### 2.12 Predicting potential drugs

The shared hub genes of psoriasis and CD were input into the Enrichr platform (https://maayanlab.cloud/Enrichr/) (Chen et al., 2013). We then utilized the Drug Signature Database (DSigDB) to identify candidate drugs associated with the hub genes (Yoo et al., 2015).

### 2.13 Simulation of molecular interactions between candidate targets and active compounds

Protein sequences were acquired from the Uniprotkb database (https://www.uniprot.org/) and then submitted to SWISS-MODEL (https://swissmodel.expasy.org/) for identification of the optimal structural template. Small molecules were searched in a small molecule database. The docking was performed using Autodock Vina 1.5.6. After completion of the docking, the conformation with the highest occurrence and the best binding effect was selected as the output result, and visual analysis was conducted using PyMOL 3.1 and DS 2019. It is widely believed that more stable ligand-receptor binding conformations correspond to lower required binding free energy. A molecular binding free energy of ≤ −5.0 kcal/mol indicates excellent binding activity (Saikia and Bordoloi, 2019).

### 2.14 Statistical analysis

The statistical analyses were carried out using GraphPad Prism (version 9.0.0.121), Python, and R (version 4.4.2). Using an unpaired Student's t-test, we compared the two groups’ gene expression in II/R damage. All analyses were considered statistically significant when the P-value was less than 0.05.

## 3 Results

### 3.1 DEGs identification

**FIGURE 2:**
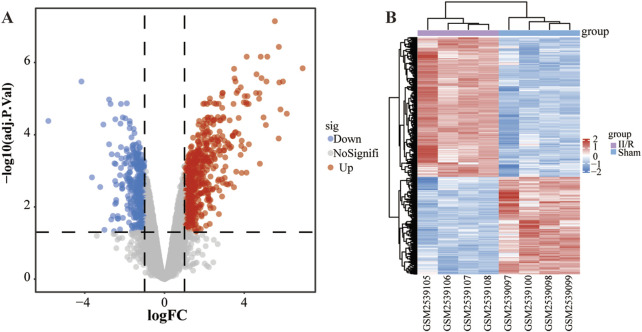
Identification of DEGs. **(A)** The volcano plot illustrates the genes that exhibit differential expression between the Sham and II/R injury samples. Red dots indicate genes that are upregulated, while blue dots correspond to downregulated genes, with established thresholds of |log2FoldChange| ≥ 1 and an adjusted *P*-value below 0.05. **(B)** The heatmap displays the differentially expressed genes between Sham and II/R injury samples. Significantly upregulated DEGs are represented in red, whereas downregulated DEGs are highlighted in blue within the samples.

Analysis using the “limma” R package identified 1,027 differentially expressed genes (DEGs) between sham-operated and II/R injury samples, among which 605 were upregulated and 422 downregulated, as visualized in Figures 2A,B. Complete results are available in Supplementary Table S1.

### 3.2 Assembly and module detection of co-expression networks

**FIGURE 3:**
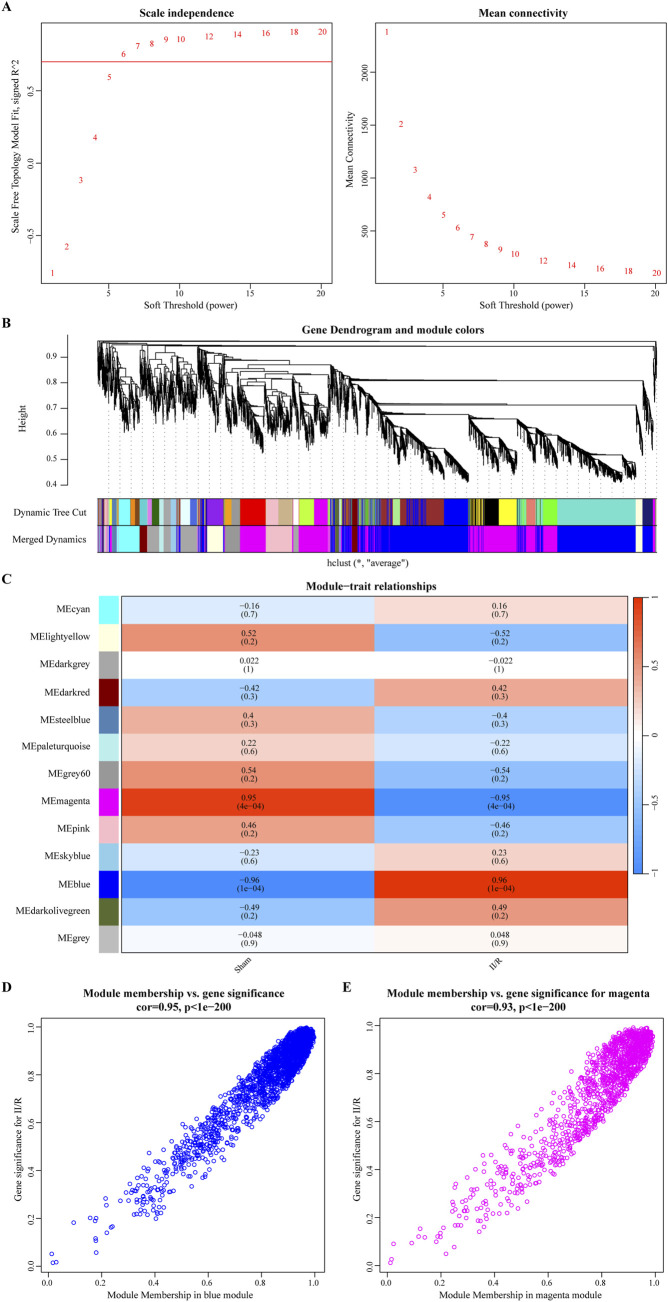
The results of WGCNA analysis. **(A)** The optimal soft threshold power is 6; **(B)** The gene clustering dendrogram; **(C)** A heatmap of the correlation between different modules and traits; **(D, E)** illustrate the scatterplot of correlations between gene significance (GS) and module membership (MM) across all modules with significant correlations.

In addition, we used WGCNA to find modular genes in the GSE96733 (II/R 3 h) dataset that are related to II/R injury. Based on the criteria of scale-free topology and average connectivity, a soft threshold power of 6 was selected, leading to the detection of 13 distinct co-expression modules (Figures 3A,C). The gray module, which lacked references and could not be assigned to any single module, was represented by this group of genes. Gene clustering results are presented in Figure 3B. According to Figure 3C, the blue and magenta modules exhibited the strongest correlation. The blue one had an absolute correlation value of 0.96, and the magenta one had 0.95. Additional evidence supporting these findings may be seen in the scatter plots of the trait association analysis for the modular genes (Figures 3D,E). Thus, employing the genes from the magenta and blue modules, we moved on to the next stage of the analysis. For information, refer to Supplementary Table S2.

### 3.3 Identification of DEMRGs

**FIGURE 4:**
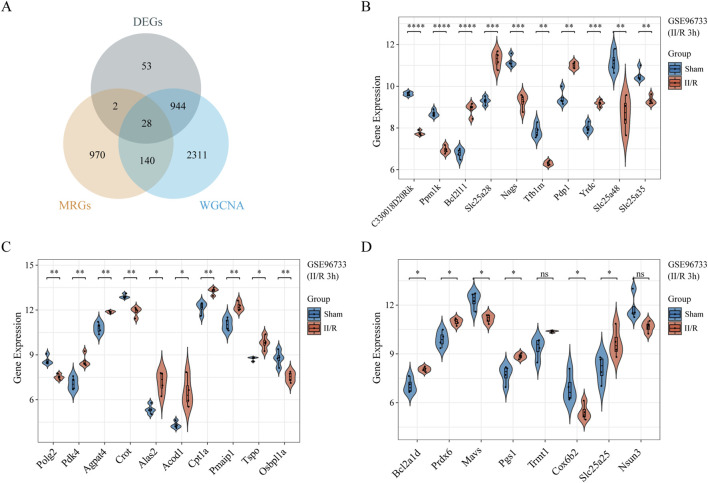
Identification of DEMRGs. **(A)** Venn diagram of DEMRGs between MRGs, DEGs, and the most relevant modules from WGCNA. **(B–D)** Violin plot illustrate the differential expression of 28 mitochondrial-related genes in both II/R and Sham samples. *P*-Values were calculated using t-test. The significance levels indicated as follows: *P < 0.05; **P < 0.01; ***P < 0.001; ****P < 0.0001. ns, not significant.

**FIGURE 5:**
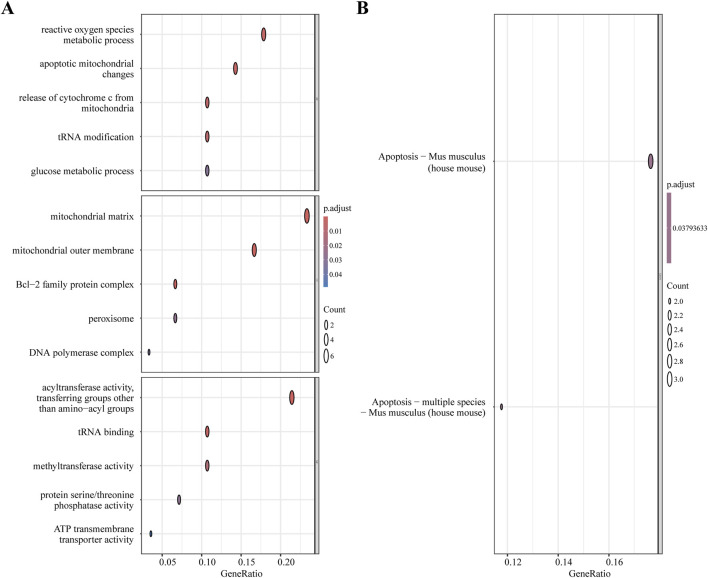
Functional enrichment analysis of DEMRGs. **(A)** GO enrichment results of DEMRGs. **(B)** KEGG enrichment results of DEMRGs.

From the MitoCarta3.0 datasets (http://www.broadinstitute.org/mitocarta), 1,140 mitochondrial-related genes (MRGs) were extracted. For the purpose of identifying 28 DEMRGs, we used the Xiantao Academic platform (https://www.xiantaozi.com/) to intersect MRGs, DEGs, and the blue and magenta modules from the WGCNA Figure 4A) Table 2 shows that out of them, 16 genes showed an increase in expression and 12 genes a decrease. Subsequently, expression profiles of the 28 DEMRGs were compared between the II/R injury and Sham groups using the “ggplot2” package in R, with results visualized via violin plots (Figures 4B–D). Results of KEGG and GO enrichment analyses for the DEMRGs, performed using a significance threshold of *P* < 0.05, are presented in Figures 5A,B. For details, refer to Supplementary Table S3.

**TABLE 2:** The gene expression of the 28 DEMRGs.

Gene symbol	logFC	T	P.Value	adj.P.Val	Changes
Bcl2l11	2.11736075	11.10621423	2.51E-07	6.57E-05	Up
Slc25a28	1.9700905	9.646193513	1.04E-06	0.000176122	Up
Pdp1	1.5590365	7.983287365	6.57E-06	0.000549823	Up
Yrdc	1.18601375	7.406222488	1.33E-05	0.00085683	Up
Pdk4	1.51492275	5.869238272	0.000106828	0.003429927	Up
Agpat4	1.0814275	5.850385755	0.000109805	0.003489425	Up
Alas2	1.8168125	5.647029097	0.000148172	0.004220079	Up
Acod1	2.18580025	5.447016037	0.000200094	0.00519498	Up
Cpt1a	1.109665	5.31584815	0.000244417	0.005980756	Up
Pmaip1	1.1964625	5.295850003	0.000252042	0.00608168	Up
Tspo	1.05966325	4.912189809	0.000459412	0.009069898	Up
Bcl2a1d	1.01812025	4.712040855	0.000633573	0.011212852	Up
Prdx6	1.072568	4.645824811	0.000705524	0.012046494	Up
Pgs1	1.114174	4.472460065	0.000937673	0.014809547	Up
Trmt1	1.00718475	3.758586109	0.003149786	0.033526877	Up
Slc25a25	1.5801765	3.447723311	0.005431908	0.048477921	Up
Nsun3	−1.170905	−3.427609303	0.00562845	0.04967905	Down
Cox6b2	−1.46816225	−3.742355899	0.003239966	0.034045771	Down
Mavs	−1.1379925	−4.5934073	0.00076856	0.012746962	Down
Osbpl1a	−1.25957875	−4.816891793	0.000535014	0.010086941	Down
Crot	−1.0126325	−5.659688091	0.000145409	0.004175612	Down
Polg2	−1.105107	−6.186788691	6.77E-05	0.002538066	Down
Slc25a35	−1.2084215	−6.468394171	4.57E-05	0.001972473	Down
Slc25a48	−2.47928175	−6.523678083	4.24E-05	0.001917251	Down
Tfb1m	−1.5443165	−8.792225562	2.59E-06	0.000313907	Down
Nags	−1.90894	−9.148338892	1.76E-06	0.000247252	Down
Ppm1k	−1.74387925	−11.55494085	1.68E-07	4.93E-05	Down
C330018D20Rik	−1.89254825	−14.12037389	2.09E-08	1.35E-05	Down

### 3.4 Building the protein-protein interaction network and pinpointing hub genes

**FIGURE 6:**
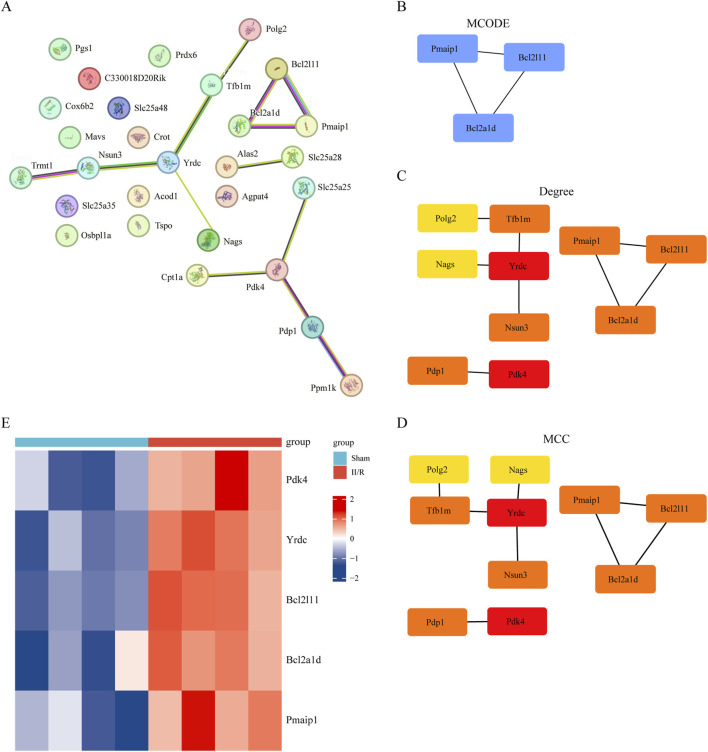
Screening of hub gene in DEMRGs. **(A)** The protein–protein interaction network visualized by STRING. **(B)** Identifying hub genes using the MCODE algorithm. **(C,D)** The PPI network constructed from 30 DEMRGs was analyzed using two algorithms: Degree, MCC. The top two genes were identified as hub genes. **(E)** The expression levels of Pmaip1, Bcl2a1d, Bcl2l11, Yrdc and Pdk4 are illustrated using a heatmap.

The protein-protein interaction (PPI) network, generated by analyzing interactions among the 28 DEMRGs in the STRING database using medium confidence settings, is displayed in Figure 6A. The network has 28 nodes and 13 edges. Figure 6B shows that we first used MCODE to choose a module that included Pmaip1, Bcl2a1d, and Bcl2l11. Afterwards, we used the CytoHubba plugin to determine the PPI network built from the 28 DEMRGs. We used two algorithms, degree and MCC, to determine which genes should be considered hub genes (Figures 6C,D). Some examples of hub genes are Yrdc, Pdk4, Bcl2a1d, Bcl2l11, and Pmaip1. Illustrating the five hub genes, Figure 6E displays their expression patterns in a heatmap. Details can be found in Supplementary Table S4.

### 3.5 Machine learning approaches were applied to screen for and confirm the final hub genes

**FIGURE 7:**
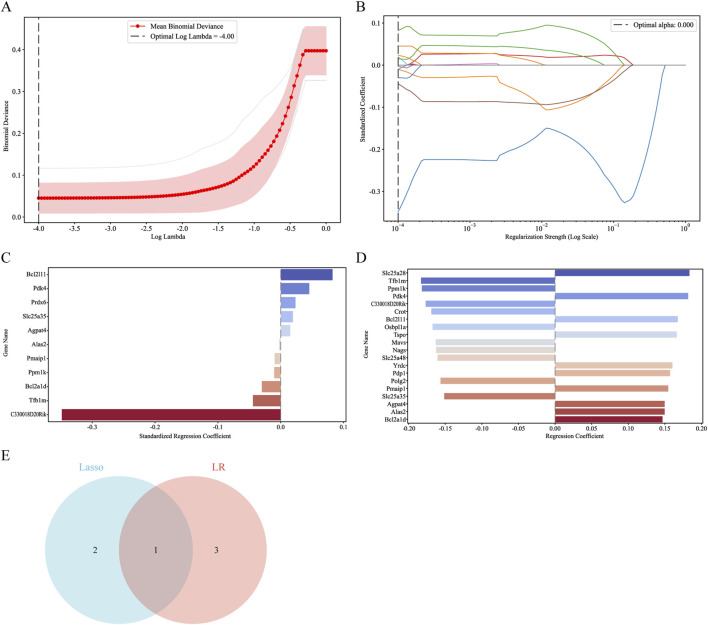
Screening for hub genes by machine learning algorithms. **(A–C)** Hub genes identified using the least absolute shrinkage and selection operator (Lasso) logistic regression algorithm. **(D)** Logistic Regression (LR) algorithm to screen hub genes. **(E)** Venn diagram demonstrating overlapping hub gene screened by Lasso and LR.

To remove hub genes from DEMRGs, we used machine learning methods. At first, we used the Lasso algorithm to pick three potential hub genes according to the requirement of |Standardized regression coefficient| ≥ 0.045 (Figures 7A–C). Afterwards, four potential hub genes were identified using the LR algorithm, and their |Regression coefficient| was 0.18 or higher (Figure 7D). We were able to identify the common gene Pdk4 (Figure 7E) using a Venn diagram. The final hub genes were determined to be Pmaip1, Bcl2a1d, Bcl2l11, Yrdc, and Pdk4 after a union with the genes chosen using the PPI network.

### 3.6 Building and evaluating the diagnostic model

**FIGURE 8:**
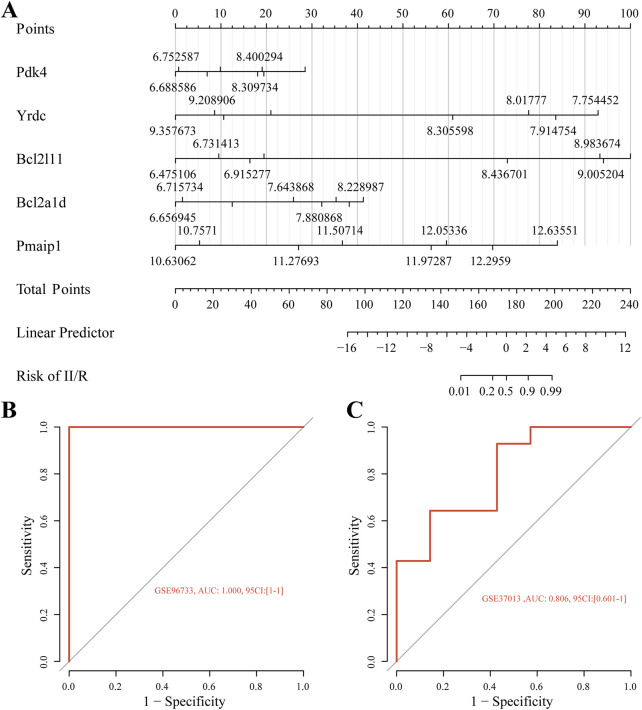
Diagnostic model construction. **(A)** Construction of a nomogram model with five hub genes. **(B,C)** ROC curve for evaluating and validating the diagnostic model’s performance.

Figure 8A shows the nomogram we developed using five identified hub genes—Pmaip1, Bcl2a1d, Bcl2l11, Yrdc, and Pdk4—as possible diagnostic indications for predicting II/R injury. Assessment of diagnostic performance using ROC curves yielded a flawless area under the curve (AUC of 1.0) for the training cohort, presented in Figure 8B. Furthermore, the GSE37013 dataset was used to validate the prediction model’s efficacy. The diagnostic model demonstrated robust predictive performance for II/R injury, achieving an AUC of 0.806 (95% CI: 0.601–1.000) (Figure 8C). Nonetheless, this diagnostic paradigm has to be further investigated in forthcoming clinical trials in order to determine its accuracy and reliability. For information, refer to Supplementary Table S5.

### 3.7 Hub gene expression patterns were validated across external datasets

**FIGURE 9:**
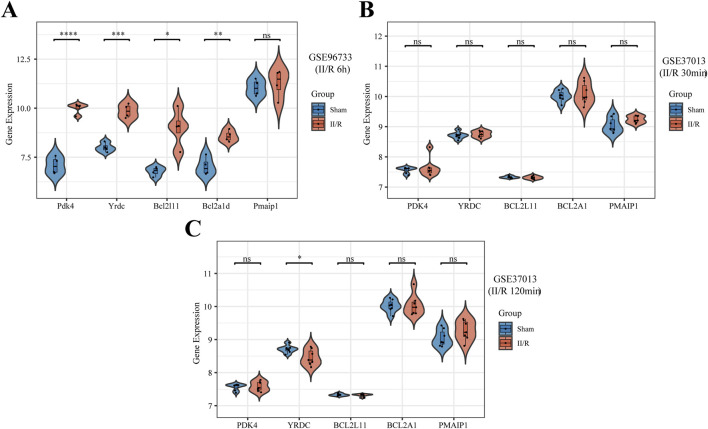
Validation of hub genes expression in other datasets. **(A–C)** The expression levels of Pmaip1, Bcl2a1d, Bcl2l11, Yrdc, and Pdk4 were verified by GSE96733 (II/R 6 h), GSE37013 (II/R 30 min), and GSE37013 (II/R 120 min) datasets, the results of which are presented as violin plot. *P*-Values were calculated using t-test. The significance levels indicated as follows: *P < 0.05; **P < 0.01; ***P < 0.001; ****P < 0.0001. ns, not significant.

As shown in Figure 9A, the expression patterns of four hub genes (excluding Pmaip1) were consistent with the bioinformatics analysis of the GSE96733 (II/R 3 h) dataset and were further validated using the GSE96733 (II/R 6 h) cohort. At 120 min after II/R damage, YRDC was still significant according to validation using the GSE37013 dataset of human injuries; expression levels were lower in the II/R group compared to the sham group (Figures 9B,C). Details can be found in Supplementary Table S6.

### 3.8 Immune cell analysis

**FIGURE 10:**
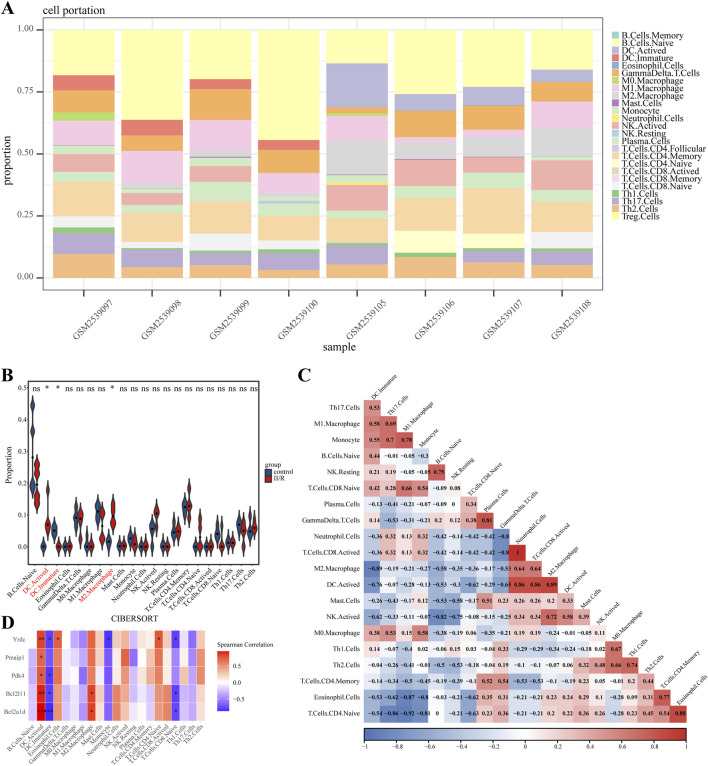
Results of immune cell analysis. **(A)** The histogram indicates the relative proportions of 25 immune cells. **(B)** Violin plot illustrating the relative expression of each immune cell subtype between Sham and II/R injury samples. **(C)** The correlation heatmap shows the relationships among various immune cells. **(D)** Heatmap showing the correlation between immune cells and five hub genes. *P < 0.05; **P < 0.01.

The CIBERSORT algorithm was employed to compare immune cell infiltration patterns between the Sham and II/R injury groups. The violin plot illustrates the variation in immune cell proportions between the two sample groups, whereas the bar graph displays the infiltration profile of specific immune cell types within individual samples. Figures 10A,B show that the II/R samples had more M2 macrophages than the Sham samples. Spearman correlation analysis revealed strong interrelationships among the various immune cell types (Figure 10C). As shown in Figure 10D, the heatmap also showed a connection between key genes and immune cell infiltration. Subtypes of immune cells infiltrating the body were substantially associated with the expression of key genes. Immature dendritic cells (DC.Immature) showed predominantly negative correlations, with the exception of a non-significant association with Pmaip1. In contrast, activated dendritic cells (DC Activated) had a positive connection with five hub genes. On the other hand, Bcl2a1d and Bcl2l11 were positively associated with M2 Macrophages. Details can be found in Supplementary Table S7.

### 3.9 RT-qPCR verification of hub genes

**FIGURE 11:**
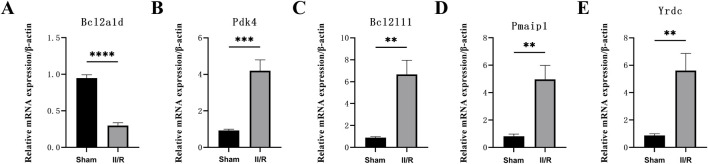
The expression levels of hub genes in the mouse II/R injury model. **(A–E)** The mRNA levels of Pmaip1, Bcl2a1d, Bcl2l11, Yrdc, and Pdk4 were assessed using qRT-PCR. *P < 0.05, **P < 0.01, ***P < 0.001, ****P < 0.0001. ns, not significant.

We used qRT-PCR on five genes associated with mitochondria to confirm the validity of the GSE96733 dataset. Yrdc, Pdk4, Bcl2l11, and Pmaip1 all exhibited significantly elevated expression in II/R injury samples relative to Sham controls, consistent with the bioinformatics predictions (Figures 11A–E). For information, refer to Supplementary Table S8.

### 3.10 Analysis of the circRNA-miRNA-mRNA regulatory network

**FIGURE 12:**
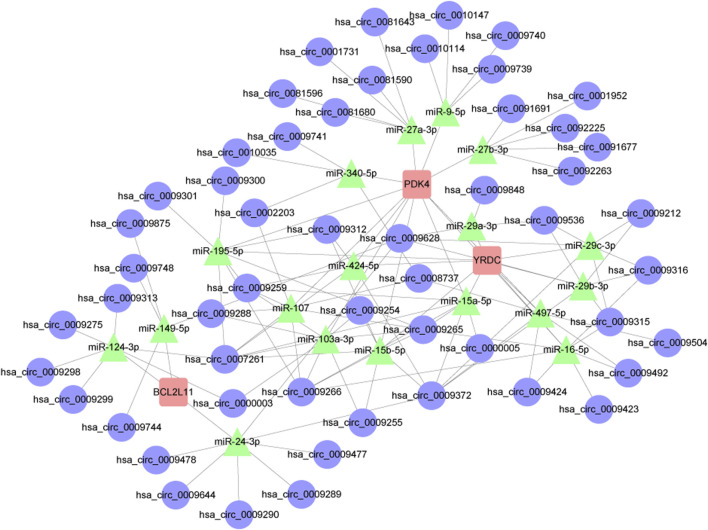
Results of the circRNA-miRNA-mRNA regulatory network. Red represents: mRNA. Green represents: miRNA. Blue represents: circRNA.

As their regulatory role in the ceRNA network becomes clear, the majority of miRNAs serve as network bridges, linking several upstream circRNAs with downstream mRNAs. Examples of circRNAs that miR-107 and miR-103a-3p can interact with include hsa_circ_0009265, hsa_circ_0009266, and hsa_circ_0009259; both can target PDK4. Furthermore, miR-124-3p binds to multiple circRNAs, including hsa_circ_0009275 and hsa_circ_0009299, indicating that it may play a key regulatory role in the apoptosis pathway as a ceRNA, in addition to targeting the apoptosis-regulating gene BCL2L11. The built network structure, consisting of numerous directed regulatory modules among circRNAs, miRNAs, and mRNAs, becomes apparent after Cytoscape display. Members of the miR-15 family (miR-15a-5p, miR-15b-5p, miR-195-5p) exhibit a typical many-to-many regulatory pattern by forming broad linkages with several target genes, including PDK4 and YRDC, and with various circRNAs. In addition to offering potential targets for verifying the involvement of non-coding RNAs in mitochondrial function during intestinal ischemia-reperfusion injury, these findings lay the theoretical groundwork for further screening of important miRNAs and the regulatory axis of their ceRNA networks (Figure 12). Details can be found in Supplementary Tables S9, S10.

### 3.11 Potential drugs prediction

**FIGURE 13:**
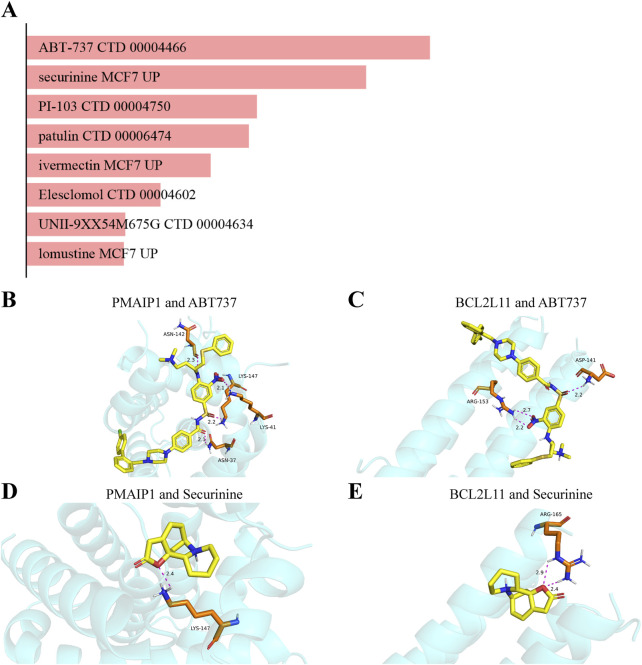
Analysis of candidate drugs and molecular docking targeting mitochondrial-related genes for the prevention of intestinal II/R injury. **(A)** Summary of candidate drugs identified from the DSigDB database. **(B,C)** Molecular docking visualization of ABT737 with BCL2L11 and PMAIP1. **(D,E)** Molecular docking visualization of Securinine with BCL2L11 and PMAIP1.

The system screens potential therapeutic medicines targeting identified hub genes by assessing the P-value and binding scores. Due to their high binding affinity and good connection with hub genes, ABT-737 and securinine have been chosen as promising potential medications following extensive testing and rectification (Figure 13A).

### 3.12 Simulating molecular interactions between candidate drugs and their protein targets

In this study, the interactions between the top two potential medications and the common proteins BCL2L11 and PMAIP1 are predicted using molecular docking analysis. Both docking activity and good docking affinity are indicated by binding energies below −6 kcal/mol and 0 kcal/mol, respectively. Figures 13B,C demonstrate that out of all the compounds examined, ABT737 had the most stable binding conformation, with binding energies of −7.1 kcal/mol to BCL2L11 and -7.9 kcal/mol to PMAIP1. Securinine also binds with BCL2L11 and PMAIP1 in a very stable conformation (−5.9 kcal/mol and −6.5 kcal/mol, respectively) (Figures 13D,E). These results are further supported by the docking conformations that may be seen. Based on these findings, ABT-737 and Securinine stand out as two potential targeted therapy meds for II/R injury.

## 4 Discussion

There are limited therapeutic options for II/R injury, despite the fact that it is a serious clinical condition (Nadatani et al., 2018). It is critically important to alleviate II/R harm through the development of novel therapeutic approaches. Mitochondria, serving as the primary organelles for cellular energy metabolism, play a critical role in the initiation and progression of II/R injury due to their functional impairments. When blood flow is interrupted, the partial pressure of oxygen in cells drops, which means that mitochondria do not take in as much oxygen. This has a knock-on effect of making oxidative phosphorylation, the main route for ATP synthesis, less efficient. In addition, intestinal oxygen consumption drops dramatically, which makes cellular functional injury worse (Archontakis-Barakakis et al., 2025). Mitochondrial malfunction in the II/R injury model could include an imbalance in the clearance of reactive oxygen species (ROS) or aberrant generation of these species (Wang F. X. et al., 2025). The inflammatory cascade response can be mediated by ROS produced from mitochondria, which can activate signaling pathways like NF-κB. This leads to the upregulation of key pro-inflammatory mediators, including TNF-α and IL-1β. Apoptosis occurs when the mitochondrial outer membrane is permeabilized and the caspase cascade is activated, for example, by caspase-3, at the same time (Cai et al., 2018; Heusch et al., 2023). Samples from GSE96733 corresponding to 45 min of intestinal ischemia followed by 3 h of reperfusion were selected for analysis. This time point was chosen due to its association with significant alterations in mitochondrial activity, consistent with prior evidence indicating early mitochondrial involvement in intestinal ischemia-reperfusion injury. There is still a lack of clarity on the connection between mitochondria and II/R injury. Not much bioinformatics research has focused on MRG expression in II/R injury. As a result, we set out to compare MRG expression in the II/R damage group to that in the control group that had sham surgery. To elucidate the immunological mechanisms underlying II/R injury and identify potential therapeutic targets, the association between immune infiltration and II/R injury was further investigated.

Using WGCNA, we initially determined which blue and magenta modules were most strongly linked to II/R injury in this investigation. The primary aim of WGCNA is to explore potential gene co-expression relationships rather than relying solely on sample size for inference. This study further integrates differential analysis, PPI networks, and machine learning to screen for key genes, and verifies the reliability of the results through external datasets and qRT-PCR experiments, thereby ensuring the scientific rigor and robustness of the analytical conclusions from multiple dimensions. Our ensuing research is built upon the 1027 DEGs and 1140 MRGs obtained from differential analysis, which we then intersected to optimize the detection of DEMRGs.

The KEGG results in the GO enrichment study of DEMRGs indicated a close link with apoptosis. The mitochondria are key regulators of cell death, according to earlier research (Jeong and Seol, 2008). GO analysis further underscores the involvement of mitochondrial processes in II/R injury pathogenesis, implicating mechanisms such as apoptosis, reactive oxygen species production, tRNA modification, and glucose metabolism. We identified DEMRGs based on these findings. Afterwards, we used the PPI interaction network, the death Lasso and LR algorithms from machine learning, and Pmaip1, as well as Bcl2a1d, Bcl2l11, Yrdc, and Pdk4 as hub genes. Analysis of the dataset revealed marked differential expression of these five hub genes between sham-operated and II/R injury groups. We used GSE96733 (II/R 6 h) to confirm these findings; Pmaip1 expression, while not statistically significant, displayed an upward trend; all other findings aligned with the initial bioinformatics results. Furthermore, a diagnostic model for II/R injury was developed and externally validated using the GSE37013 dataset. It proved to be quite effective in making diagnoses.

One method that predicts the relative number of immune cells in mixed cell populations by examining gene expression data is CIBERSORT, an inverse convolution analysis algorithm based on linear support vector regression (Newman et al., 2015). Hub gene correlation analysis revealed higher abundances of activated dendritic cells (DC. Activated) and M2 macrophages in II/R injury samples compared to sham controls. Actived had a positive correlation with five hub genes, whereas DC. Immature had a negative correlation with all save Pmaip1. There was a positive link found between M2 Macrophages and Bcl2a1d and Bcl2l11. A number of immune cells seem to have strong ties to one another, according to the correlation study.

As members of the innate immune system, macrophages are very adaptable and can change their appearance in response to outside influences. Macrophages safeguard the intestinal barrier when homeostasis is achieved (Ruder and Becker, 2020). In a previous study, we found that encouraging M1 macrophages to convert to M2 could lessen II/R injury (Liu et al., 2015). New evidence suggests that II/R injury can be mitigated by stimulating M2 macrophage interleukin-10 release through Toll-like receptor 2 signaling (Hu et al., 2022). By controlling macrophage M1 polarization and mitochondrial abnormalities, Germacrone ameliorates acute lung injury brought on by intestinal ischemia-reperfusion, according to research (Wang et al., 2024). There has to be further research to corroborate the rumor that Nrf2 regulates macrophages and mitochondrial activity, which could improve II/R injury. An integral part of the intestinal immune system’s regulatory machinery are dendritic cells (DC). DCs are more likely to infiltrate the small intestine after II/R damage, and TLR4 is upregulated as a result (Hagiwara et al., 2010). This finding provides more evidence that TLR4 is essential for II/R damage autophagy and immune cell modulation. Theoretically, this lays the groundwork for novel therapeutic approaches to II/R injury.

Lastly, *in vitro* studies were conducted to examine the spatial or temporal distribution of hub genes in a murine II/R injury model. RT-qPCR results demonstrated markedly distinct expression profiles of Pmaip1, Bcl2l11, Yrdc, and Pdk4 between II/R injury and sham groups. These findings align with earlier bioinformatic predictions of analogous expression trends.

Phorbol-12-myristate-13-acetate-induced protein 1 (Pmaip1), a BCL-2 protein is essential to apoptosis, regeneration and disease pathways (Morsi et al., 2018).In an animal model of myocardial infarction/reperfusion, Pmaip1 and BimEL protein levels were elevated after 3 hours of reperfusion. These two proteins counteract Mcl-1, which collapses mitochondrial membrane potential (Ishihara and Shimamoto, 2012). This releases cytochrome c into the cytoplasm. This indirectly suggests that Pmaip1 regulates mitochondrial apoptosis to alleviate intestinal ischemia-reperfusion injury. Although Pmaip1 expression in the GSE96733 (II/R 6 h) validation cohort did not reach statistical significance, its upward trend was consistent with the overall expression pattern observed in the GSE96733 (II/R 3 h) dataset and qRT-PCR validation. This temporal difference likely reflects the transient activation of Pmaip1-mediated mitochondrial apoptosis during the early phase of intestinal ischemia–reperfusion injury.

In this study, Bcl2a1d was found to be upregulated in bioinformatics analysis, while qRT-PCR results indicated downregulation, suggesting that its anti-apoptotic function is inhibited during intestinal ischemia-reperfusion injury. As a member of the Bcl-2 family, Bcl2a1d normally maintains cell survival by stabilizing mitochondrial membrane potential and inhibiting the release of cytochrome c (Sugioka et al., 2003). The intense inflammatory response and oxidative stress following reperfusion may weaken its anti-apoptotic capacity, leading to decreased expression and thereby promoting apoptosis and tissue damage. Currently, research on Bcl2a1d is still limited, and further studies are needed to support these findings.

Bim, encoded by Bcl2l11, is a BH3-only protein that can cause cell death by inhibiting BCL2, a protein that suppresses cell death, or by directly activating BAX and BAK1, 2 cell death effectors (Luo and Rubinsztein, 2013). BATF transcription factor suppresses Bim expression to prevent T cell apoptosis. Bim overexpression in BATF-deficient cells increases T cell mortality and decreases PD-1+ and Treg populations, suggesting Bcl2l11 is implicated in immunity (Titcombe et al., 2023). In hypoxia/reoxygenation (H/R) damage, the FOXO3a transcription factor brings the pro-apoptotic protein Bim to the mitochondria. This collapses the mitochondrial membrane potential, opens the mPTP, releases cytochrome c, and activates the caspase cascade.

Neuronal cells can be protected from Bim buildup using mitochondrial transplantation therapy, which activates the FUNDC2/PIP3/Akt/FOXO3a axis (Shi et al., 2021). These findings further point to the possibility that Bcl2l11 regulates apoptosis, which in turn improves mitochondrial activity and reduces intestine ischemia-reperfusion injury.

One of the most conserved protein families, Yrdc is involved in ribosome maturation and other important cellular biological processes. it controls the processing of 16S rRNA in order to guarantee the correct assembly of ribosomes (Kaczanowska and Rydén-Aulin, 2004; 2005). In addition, it is an essential component of N6-threonylcarbamoyladenosine and an essential member of the YrdC/Sua5 family that The process of (t6A) alteration in tRNA is crucial for preserving genomic stability and guaranteeing accurate translation (Wan et al., 2013).

Pdk4 is a mitochondrial pyruvate dehydrogenase complex (PDC) negative regulatory kinase that phosphorylates PDC to restrict its activity and decrease pyruvate to acetyl-CoA conversion (Kukimoto-Niino et al., 2011). Inhibiting PDC is one way that Pdk4 controls mitochondrial energy metabolism. An aberrant buildup of succinate within the mitochondria occurs when Pdk4 is activated during ischemia periods. During the reperfusion phase, the built-up succinate causes an overproduction of reactive oxygen species (ROS) by the mitochondria, which in turn causes severe damage to the mitochondria and worsens the tissue injury produced by ischemia-reperfusion (Li et al., 2017; Nishima and Tanaka, 2023; Oh et al., 2023; Khang et al., 2024).

There is a growing body of literature linking II/R damage to non-coding RNAs. Studies have shown that the inhibition of miR-379-5p can enhance epithelial cell proliferation and improve barrier function after II/R injury (Jia et al., 2023). Due to its anti-inflammatory and anti-apoptotic capabilities, suppressing miR-665-3p could be a promising clinical treatment for II/R damage (Li et al., 2018). MiR-26b-5p has the potential to reduce apoptosis in intestinal mucosal cells and target DAPK1, therefore preventing II/R injury (Zhou et al., 2022). Based on our findings, miR-124-3p may serve as a central regulator in the ceRNA network associated with the apoptosis pathway, since it targets the apoptosis-related gene BCL2L11 and interacts with multiple circRNAs, including hsa_circ_0009275 and hsa_circ_0009299. The miR-15 family (miR-15a-5p, miR-15b-5p, miR-195-5p) has a typical multi-to-multi regulation pattern with broad links to numerous target genes, including PDK4 and YRDC, and other circRNAs. These results provide a fresh angle on II/R injury research by providing theoretical groundwork for the future discovery of important miRNAs and the ceRNA regulatory axes that regulate them. They also suggest potential targets for further validation of the role of non-coding RNAs in mitochondrial function during II/R injury.

An inhibitor of Bcl-2 family proteins, ABT-737 is a small chemical BH3 mimic (Guan et al., 2021). Research found that combining arsenic trioxide with ABT-737 can enhance mitochondrial apoptosis and downregulation of Mcl-1 in cervical cancer cells, leading to a synergistic lethal impact (Hsin et al., 2019). There is a very stable binding conformation between ABT-737 and BCL2L11 since the binding energy is comparatively low (−7.1 kcal/mol). Shorter hydrogen bonds are associated with reduced energy and higher system stability, while the typical range for hydrogen bond lengths is 1.5–3.5 Å. With hydrogen bond lengths of 2.2 Å and 2.7 Å, respectively, ABT-737 and BCL2L11 are stable. Despite the lack of ABT-737s use in II/R injury treatment thus far, this is an innovative aspect that opens up new possibilities. In 1956, an alkaloid called securinine was discovered and identified from the plant Securinega suffruticosa (Pall.). Roots of plants from the Phyllanthus and Flueggea genera, as well as those of plants in the Securinega genus, contain it, according to research (Hou et al., 2023). Securinine inhibits tumor growth primarily through activation of the mitochondrial apoptotic pathway. In DU145 prostate cancer cells, it upregulates Bax expression, facilitates cytochrome c release, and initiates the caspase-9/caspase-3 signaling cascade. Concurrent downregulation of the anti-apoptotic protein Bcl-2 further confirms its reliance on the intrinsic mitochondrial mechanism to trigger programmed cell death (Zhang et al., 2019). The binding conformation of securinine with BCL2L11 is also highly stable (−5.9 kcal/mol). The hydrogen bonds between securinine and BCL2L11 are measured at 2.4 Å and 2.9 Å, indicating a similar level of stability. This also provides evidence for securinine’s role in improving II/R injury through mitochondrial regulation. ABT-737, as a BCL-2 family inhibitor, exhibits significant pro-apoptotic effects, and its presence in this study is based solely on the computational results from molecular docking and database screening. Given that one of the core pathological mechanisms of intestinal ischemia-reperfusion injury is excessive apoptosis, the application of this drug in this context may pose potential risks. Therefore, the predictive results for ABT-737 and similar compounds should not be regarded as direct therapeutic recommendations but rather understood as theoretical molecular interaction clues. Future studies will further validate the actual mechanisms of action of these candidate drugs in cellular protection or damage through experimental methods, in order to assess whether they may exert regulatory or protective effects under specific dosage, timing, or target conditions, thus providing a basis for safer and more rational therapeutic strategies.

There are inevitably certain limitations in this study. Firstly, the diagnostic model established in this research demonstrated a very high predictive accuracy in the training set (AUC = 1.0), suggesting a potential degree of overfitting. This result is closely related to the limited sample size, as GSE96733 only includes 8 samples, making it difficult to fully represent the molecular heterogeneity of intestinal ischemia-reperfusion injury. Although we performed dual feature selection using LASSO and logistic regression and validated the model with the external dataset GSE37013 (AUC = 0.806), it must be acknowledged that the generalizability of the model may be constrained. Future research will aim to enhance the model’s stability and clinical applicability by increasing the sample size, introducing multi-time points, multi-species, and clinical sample validations. Secondly, the temporal analysis of gene sets has not covered the dynamic changes in the later stages of reperfusion (>6 h), while clinical observations indicate that intestinal barrier dysfunction has a delayed effect, necessitating an extension of the observation time window in subsequent studies. This study selects 3 h of reperfusion as the primary observation time point, aiming to focus on the early mitochondrial dysfunction and related molecular mechanisms following II/R injury. It is noteworthy that clinical studies indicate that the damage to the intestinal barrier function often lags behind the early stage of mitochondrial injury, typically requiring a longer duration to manifest at the tissue structural and functional levels. Therefore, future research could extend the reperfusion time (e.g., 6 h, 12 h) and combine multi-omics and histological analyses to comprehensively reveal the dynamic relationship between early molecular changes and subsequent tissue damage. Furthermore, RT-qPCR only validated the mRNA levels of hub genes in the mouse intestinal ischemia-injury model. More relevant *in vivo* and *in vitro* experiments are needed to demonstrate the roles of these hub genes and their potential mechanisms in ischemia-reperfusion injury, exploring Pmaip1, Bcl2l11, Yrdc, and Pdk4 as regulators of mitochondria to improve intestinal ischemia-reperfusion injury and promote translational applications.

## 5 Conclusion

Employing bioinformatics methods, this study investigated the association between early II/R injury and mitochondria-related genes, identifying four central genes: Pmaip1, Bcl2l11, Yrdc, and Pdk4. These genes may serve as potential biomarkers for detecting II/R injury, elucidating its mechanisms, and guiding therapeutic strategies. Additionally, ABT-737 and securinine were predicted as promising drug candidates. A circRNA–miRNA–mRNA regulatory network centered on these hub genes was constructed, and immune infiltration patterns were analyzed. These results provide a foundation for future drug development and clinical research in II/R injury.

## Data Availability

The datasets presented in this study can be found in online repositories. The names of the repository/repositories and accession number(s) can be found in the article/Supplementary Material.
